# Rosemary Diterpenes and Flavanone Aglycones Provide Improved Genoprotection against UV-Induced DNA Damage in a Human Skin Cell Model

**DOI:** 10.3390/antiox9030255

**Published:** 2020-03-20

**Authors:** Noelia Sánchez-Marzo, Almudena Pérez-Sánchez, Enrique Barrajón-Catalán, Julián Castillo, María Herranz-López, Vicente Micol

**Affiliations:** 1 Instituto de Investigación, Desarrollo e Innovación en Biotecnología Sanitaria de Elche (IDiBE), Instituto de Biología Molecular y Celular (IBMC), Miguel Hernández University (UMH), 03202 Elche, Spain; n.sanchez@umh.es (N.S.-M.); almudena.perez@umh.es (A.P.-S.); mherranz@umh.es (M.H.-L.); vmicol@umh.es (V.M.); 2 Nutrafur S.A., Camino Viejo de Pliego, km.2, 30820 Alcantarilla, Murcia, Spain; j.castillo@nutrafur.com; 3 Department of Food Technology and Nutrition, Universidad Católica San Antonio, 30107 Guadalupe de Maciascoque, Murcia, Spain; 4 CIBER, Fisiopatología de la Obesidad y la Nutrición, CIBERobn, Instituto de Salud Carlos III (ISCIII) (CB12/03/30038), 28029 Madrid, Spain

**Keywords:** rosemary, diterpene, flavone, natural extract, cosmetic, ROS, antioxidant

## Abstract

Overexposure to solar ultraviolet (UV) radiation is the major cause of a variety of cutaneous disorders, including sunburn, photoaging, and skin cancers. UVB radiation (290–320 nm) causes multiple forms of DNA damage, p53 induction, protein and lipid oxidation, and the generation of harmful reactive oxygen species (ROS). In recent years, botanicals containing polyphenols with antioxidant and anti-inflammatory properties as skin photoprotective agents have emerged. This study evaluated the protective effects of two formulations against UVB-induced damage in a skin cell model. One of the formulations (F2) contained a combination of citrus and olive extracts and the other one (F1) also contained a rosemary extract. The antioxidant capacity of both formulations was estimated by different in vitro methods, and the cell viability, intracellular ROS generation, mitochondrial depolarization, and DNA damage were studied in UVB-irradiated human keratinocytes. Both formulations exerted photoprotective effects on skin cells and decreased mitochondrial depolarization and DNA damage. F1 which contained iridoids, rosemary diterpenes, glycosides and aglycones of citrus flavanones, and monohydroxylated flavones exhibited higher cellular photoprotective effects and mitochondrial membrane potential restoration, as well as an enhanced capacity to decrease DNA double strand breaks and the DNA damage response. In contrast, F2, which contained mostly iridoids, citrus flavanone aglycones, and mono- and dihydroxylated flavones, exhibited a higher capacity to decrease intracellular ROS generation and radical scavenging capacity related to metal ion chelation. Both formulations showed a similar capability to decrease the number of apoptotic cells upon UVB radiation. Based on our results and those of others, we postulate that the stronger capacity of F1 to protect against UVB-induced DNA damage in human keratinocytes is related to the presence of rosemary diterpenes and citrus flavanone aglycones. Nevertheless, the presence of the dihydroxylated flavones in F2 may contribute to inhibiting the generation of metal-related free radicals. To confirm the efficacy of these formulations as potential candidates for oral/topical photoprotection, human trials are required to circumvent the limitations of the cellular model.

## 1. Introduction

The skin is the largest organ of the human body, accounting for approximately 15% of total body weight, and it performs vital functions such as thermoregulation and protection against external agents [1]. Solar ultraviolet (UV) radiation is the most prominent physical carcinogen in our natural environment and is known to have several harmful effects, including erythema, edema, sunburn cells, hyperplasia, immunosuppression, premature aging, and photocarcinogenesis [2].

UVA (320–400 nm) accounts for 95% of all solar UV radiation that arrives at the Earth’s surface and plays a crucial role in photoaging, while UVB (280–320 nm) is mainly responsible for sunburn and skin cancers. Both UVA and UVB contribute to reactive oxygen species (ROS) generation and oxidative stress [3]. These ROS, including superoxide anion radicals (O_2_^•−^), hydroxyl radicals (^•^OH), and their active precursors, namely, singlet oxygen (^1^O_2_), hydrogen peroxide (H_2_O_2_), and ozone (O_3_), cause not only protein oxidative damage but also lipid peroxidation, producing peroxyl radicals, which damage cell membranes [4]. Subsequently, the cell membrane fluidity is decreased, and the mitochondrial membrane is depolarized, which could compromise adenosine triphosphate (ATP) formation and energy homeostasis, leading to extensive cellular damage or death. ROS also target guanine DNA bases, giving rise to 8-hydroxydeoxyguanosine (8-OHdG), a ubiquitous marker of oxidative stress. Cyclobutane pyrimidine dimers (CPDs), a major class of DNA photolesions, are mostly induced by UVB radiation, but these lesions also seem to be significantly produced in human skin exposed to UVA [5]. Incorrect repair of these lesions can result in mutated oncogenes and tumor suppressor genes, since p53 mutation is found in more than fifty percent of all nonmelanoma skin cancers (NMSCs), which is the most common class of malignant neoplasms with increased incidence due to greater UV exposure [6]. Furthermore, UV radiation also induces DNA double-strand breaks (DSBs), which are probably the most dangerous damage to DNA because they could lead to the formation of chromosome aberrations [7]. Direct DNA damage, oxidative stress, and the activation of cell surface receptors induce apoptosis in severely damaged cells as a protective mechanism, reducing the risk of malignant transformation. Sunburn cells (SCs) are single standing cells with typical morphologic features that are detectable 8 h after UV exposure with maximum prevalence after 24–48 h. Microscopic and ultrastructural studies of SCs has allowed their recognition as keratinocytes undergoing apoptosis [8]. The natural pigment melanin absorbs and scatters UV radiation, acting as the major protective barrier of the skin [9]. Moreover, enzymatic and nonenzymatic antioxidant arsenals within skin cells protect against UV-induced oxidative damage, but this may not be sufficient to counteract excessive pro-oxidant insults.

UVB is also known to upregulate gene expression through intracellular signal transduction pathways related to inflammation, cell survival, and proliferation. Alterations in the NF-κB, mitogen-activated protein kinase (MAPK), phosphoinositide-3 kinase (PI3K), and extracellular signal-regulated kinase (ERK1/2) pathways have been found in UV-irradiated cells, and these changes may contribute to the development of skin cancer [10,11]. Photoaging is another harmful effect of UVB exposure that has been extensively studied. Although UVA radiation is considered the main factor responsible for premature aging, UVB light enhances the expression of interstitial collagenase and stromelysin-1, the two major members of the matrix metalloproteinase family, resulting in a dramatic decrease in collagen and an overgrowth of abnormal elastic fibers [12].

A wide variety of phytochemicals has shown potential health benefits, and their use as photoprotective compounds has gained considerable attention. Plant polyphenols possess strong free radical scavenging abilities and reduce UV-induced oxidative damage. The effects of resveratrol, a polyphenol present in grapes and red wine, and the effects of green tea polyphenols have been well studied [13,14]. Numerous flavonoids, such as apigenin, genistein, and quercetin, protect against UV-induced oxidative stress [15,16,17]. Furthermore, plant terpenoids also act as strong antioxidant compounds, and both polyphenols (especially flavonoids and isoflavones) and terpenoids display anti-inflammatory and antiphotocarcinogenic effects by modulating cell signaling pathways and regulating the cell cycle [18,19]. Several botanical extracts have exhibited a protective effect against UV radiation, such as *Aloe vera*, *Punica granatum,* and *Silybum marianum* [20,21,22]. In addition, different human trials have used nutraceutical products based on botanical extracts to show their effects on skin health [23]. We recently demonstrated a higher protective effect for a *Melissa officinalis* extract than for its major phenolic compound (rosmarinic acid) against UVB-induced damage [24]. The synergistic photoprotective effect of rosemary and citrus extracts in vitro and in vivo has also been reported [25].

**Figure 1:**
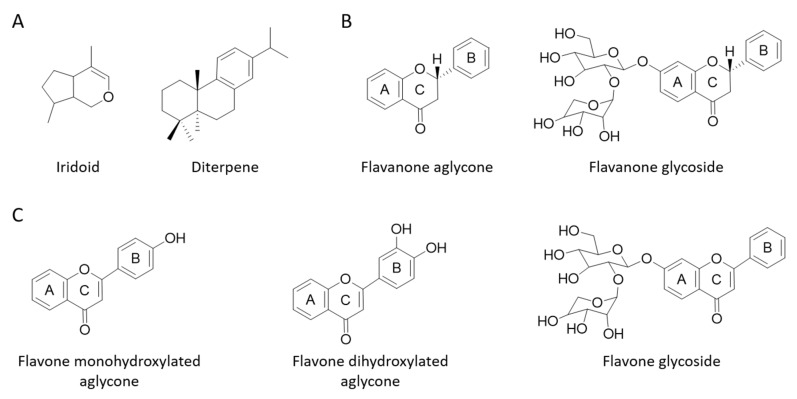
General structure of the phytocompounds families that were present in the studied formulations grouped into terpenes (**A**), flavanones (**B**), and flavones (**C**).

In the present study, two specific botanical combinations containing citrus, olive, and rosemary extracts were used to inhibit the harmful effects of UVB in a skin cell model. Figure 1 shows the general structure of the phytochemicals declared by the manufacturer in the formulations. The capacity of both formulations to decrease UVB-mediated cell death, ROS formation, mitochondrial depolarization, and DNA damage was assessed and compared in human keratinocytes.

## 2. Materials and Methods

### 2.1. Materials

Human keratinocytes (the spontaneously immortalized cell line HaCaT) were obtained from Cell Lines Service (CLS) GmbH (Eppelheim, Germany). Dulbecco’s Modified Eagle’s Medium (DMEM), fetal bovine serum (FBS), and penicillin-streptomycin were purchased from Gibco™/Thermo Fisher Scientific (Waltham, MA, USA). Dimethyl sulfoxide (DMSO) and the rest of the reagents were purchased from Sigma-Aldrich (St. Louis, MO, USA). Botanical formulations were kindly provided by NUTRAFUR, S.A. (Alcantarilla, Murcia, Spain).

### 2.2. Formulations

The two formulations were composed of a flavonoid-enriched citrus extract and an olive extract containing iridoids as declared by the manufacturer. Formulation 1 (F1) also contained a rosemary extract rich in polyphenols and diterpenes, as declared, while formulation 2 (F2) was composed exclusively of citrus and olive compounds. The relative percentages of flavonoid (flavanone and flavone), iridoid, and diterpene dry weights are detailed in Table 1. Both formulations were dissolved in phosphate-buffered saline (PBS) and DMSO (50:50) and were freshly prepared for each assay.

### 2.3. Total Phenolic Content Determination and Absorption Spectra

The total phenolic content was quantified according to the Folin-Ciocalteu method using gallic acid as a reference standard phenol, and the results are expressed as gallic acid equivalents (GAE)/100 g dry weight (dw) [26,27]. Absorption spectra collection was performed on a microplate reader (SPECTROstar Omega, BMG LabTech GmbH, Germany). Both formulations were prepared at different concentrations (50 and 100 µg/mL), and the absorbance was measured in the range from 245 to 600 nm at 4 nm intervals with three replicates at each point.

### 2.4. In Vitro Antioxidant Activity Assays

The antioxidant capacity of both formulations was determined by performing three different in vitro methods. The Trolox equivalent antioxidant capacity (TEAC) assay was performed as previously described to measure the ABTS^•+^ (2,2′-azino-di-[3-ethylbenzothiazoline-6-sulfonic acid] radical cation) scavenging ability of F1 and F2 [28]. The results are expressed in mmol equivalents of Trolox (TE)/100 g dw. The oxygen radical absorbance capacity (ORAC) assay was performed as described elsewhere to evaluate antioxidant inhibition of peroxyl radical-induced oxidation using fluorescein [29]. The final ORAC values were calculated from the net area under the fluorescence decay curves (AUC) and are reported as µmol TE/g dw. The ferric reducing ability power (FRAP) assay was performed essentially as previously described to estimate the reduction of a ferric-tripyridyltriazine complex [30]. The results are expressed in mmol equivalents Fe^2+^/100 g dw.

### 2.5. Maintenance and Treatment of the Keratinocyte Cell Culture

HaCaT cells were grown in DMEM supplemented with 10% (*v*/*v*) FBS and 1% (*v*/*v*) penicillin-streptomycin (0.1 mg/mL penicillin and 100 U/mL streptomycin) at 37 °C in a humidified 5% CO_2_ atmosphere. The cell culture was trypsinized every third day, following the manufacturer’s instructions.

Prior to UVB irradiation, the cells were cultured in 96- or 6-well plates, depending on the assay, and were maintained in medium for 24 h. When 70–80% confluence was reached, cells were washed with PBS and treated with the formulation (F1 or F2) dissolved in a thin layer of PBS, followed by UVB light treatment emitted from a Bio-Link Crosslinker BLX-E312 (Vilber Lourmat, France). In parallel, nonirradiated cells were treated similarly to evaluate the toxicity of the formulations. Subsequently, cells were washed with PBS and incubated with fresh medium for 24 h prior to analysis of the following parameters: cell survival, mitochondrial depolarization, apoptotic cell death, H2AX histone activation, and DNA double strand breaks.

The percentage of protection from all assays was calculated as the percent recovered under a certain condition using the following formula, where 100% was the difference between the nonirradiated, nontreated cells (PC) and the irradiated cells in the absence of the extract (NC):

(1) $$Protection\;\left(\%\right)=100-100\times \left(\frac{PC-sample}{PC-NC}\right)$$

### 2.6. Cell Survival Quantitation

For the survival assay, cells cultured in 96-well black plates were treated with F1 or F2 (50–200 µg/mL) and UVB light treatment (800 or 1200 J/m^2^). Survival quantitation was determined by nuclear staining using the Hoechst 33342 fluorescent probe (Molecular Probes™, Invitrogen™/Thermo Fisher Scientific, Waltham, MA, USA). Cells were incubated with the probe for 30 min, and the fluorescence was measured using a Cytation 3 Cell Imaging Multimode reader (BioTek, Winooski, VT, USA) with 377 nm excitation and 447 nm emission filters.

### 2.7. Mitochondrial Depolarization Evaluation

The mitochondrial membrane potential (MMP) was analyzed using two fluorescent dyes: MitoTracker Red CMXRos, whose fluorescence is dependent on the MMP, and MitoTracker Green FM, which stains mitochondria independent of the MMP for normalization (Molecular Probes™, Invitrogen™/Thermo Fisher Scientific, Waltham, MA, USA). HaCaT cells seeded in a 96-well black plate that were previously treated with F1 or F2 (50–200 µg/mL) and UVB irradiation (1200 J/m^2^) were incubated with both fluorescent dyes (200 nM) for 30 min. Fluorescence was measured using a Cytation 3 Cell Imaging Multimode reader with 490 nm excitation and 520 nm emission filters for MitoTracker Red and 574 nm excitation and 604 nm emission filters for MitoTracker Green.

Mitochondrial depolarization was also explored using flow cytometry. To analyze the changes in the cellular MMP, a Muse MitoPotential Assay Kit (Merck Millipore, Burlington, MA, USA) was used following the manufacturer’s directions with trypsinized cells, and population profiles were obtained using the Muse Cell Analyzer (Merck Millipore, Burlington, MA, USA). Formulations (100 or 200 µg/mL) and UVB light (1200 J/m^2^) treatments were previously performed in HaCaT cells cultured in 6-well plates.

### 2.8. Apoptotic Cell Death and Detection of H2AX Activation

The percent apoptotic cells and H2AX histone activation were determined by the Muse Cell Analyzer (Merck Millipore, Burlington, MA, USA). Apoptotic cell death was evaluated using trypsinized culture and the Muse Annexin V and Dead Cell Kit (Merck Millipore, Burlington, MA, USA) according to the manufacturer’s instructions, while the Activation Dual Detection Kit (Merck Millipore, Burlington, MA, USA) was employed for the detection of the phosphorylated (activated) form of the H2AX histone protein (γ-H2AX, a marker of DNA damage). For both assays, HaCaT cells were previously cultured in 6-well plates and treated with the formulations (100 or 200 µg/mL) and UVB irradiation at a dose of 1200 J/m^2^.

### 2.9. DNA Double Strand Breaks in Single-Cell Gel Electrophoresis (Comet Assay)

For the comet assay, neutral single-cell gel electrophoresis (SCGE) was essentially performed in duplicate as previously reported to detect DNA double strand breaks (DSBs) [31]. Six-well plate-seeded cells were treated with 200 µg/mL F1 or F2 and UVB irradiation and were incubated for 24 h in fresh medium. Thereafter, the cells were trypsinized, pelleted, resuspended in PBS, and mixed with low melting point agarose (0.7%). A total of ≥100 nuclei were utilized for measuring various SCGE parameters (i.e., head and tail DNA content, tail length, tail migration, and tail moment) using image analysis software (Comet assay IV, Perceptive Instruments, UK). The tail moment value (product of the tail length and tail DNA content) was selected for representation, as it is the most frequent parameter used for UV-protective studies in the literature [32].

### 2.10. Intracellular ROS Generation Measurement

The intracellular ROS generation induced by UVB radiation was monitored using the 2′,7′-dichlorodihydrofluorescein diacetate (H_2_DCF-DA) fluorescent probe (Molecular Probes™, Invitrogen™/Thermo Fisher Scientific, Waltham, MA, USA). HaCaT cells were cultured in 96-well black plates for 24 h and were treated with the formulations (100 or 200 µg/mL) and UVB irradiation (800 or 1200 J/m^2^). In this case, cells were incubated with H_2_DCF-DA and Hoechst 33342 for 30 min immediately after irradiation. Fluorescence was measured using a Cytation 3 Cell Imaging Multimode reader with 485 nm excitation and 535 nm emission filters for H_2_DCF-DA, while Hoechst 33342 was detected with the filters mentioned above. The fluorescence signal of the probe in each well was normalized using the number of nuclei.

### 2.11. Statistical Analysis

The data were analyzed by GraphPad Prism version 6.01 (GraphPad Software, San Diego, CA, USA) and are expressed as the mean ± standard deviation (SD) of 5–16 determinations, depending on the assay. The obtained values for the two formulations from the Folin-Ciocalteu and *in vitro* antioxidant activity assays (TEAC, ORAC, FRAP) were compared through Student’s t-test, while one-way ANOVA and statistical comparisons of the different treatments using Tukey’s test were performed for the cellular experiments. Statistical differences were considered to be significant at *p* < 0.05. * *p* < 0.05, ** *p* < 0.01, *** *p* < 0.001 and **** *p* < 0.0001 in the figures indicate statistically significant differences compared to the irradiated control. # *p* < 0.05, ## *p* < 0.01, ### *p* < 0.001 and #### *p* < 0.0001 indicate statistically significant differences between treatments with each formulation at the same concentration.

## 3. Results

### 3.1. Photoprotective Effects of the Formulations on the Viability of HaCaT Cells Exposed to UVB Irradiation

**Figure 2:**
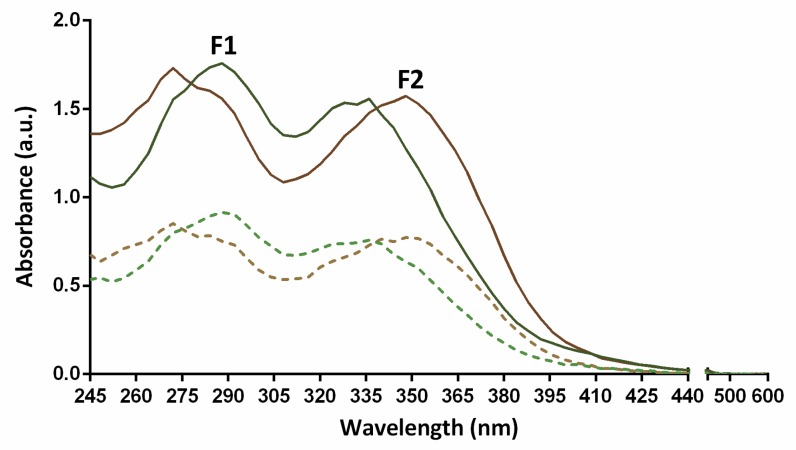
Absorption spectra of the formulations F1 and F2 from 245 to 600 nm. Continuous lines indicate formulation absorption spectra at 100 µg/mL while dashed lines show absorption spectra obtained with 50 µg/mL of formulation.

Cell viability was determined through nuclear staining after UVB irradiation (800 or 1200 J/m^2^ dose) to evaluate the photoprotective effects of the formulations F1 and F2. Previously, the absorption spectra of the formulations were measured (Figure 2), showing a significant absorption in the range of UVB spectra (280–320 nm).

**Figure 3:**
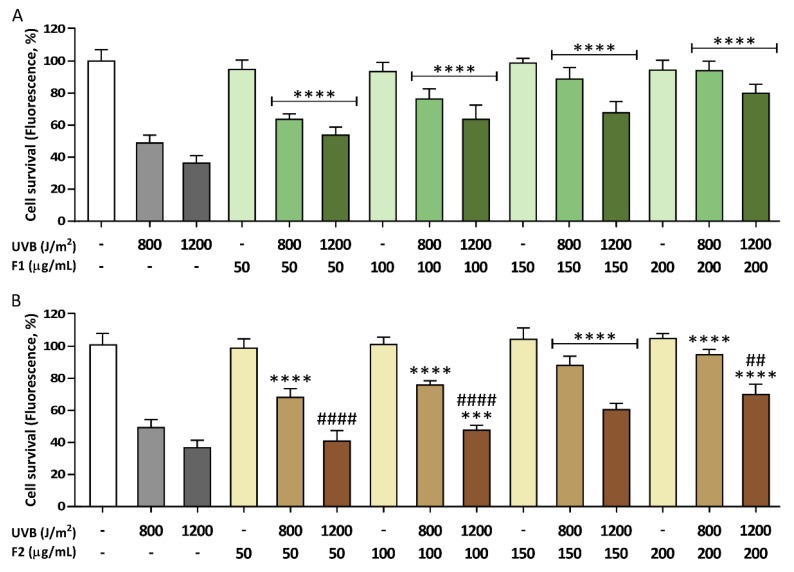
Photoprotective effects of formulations F1 (**A**) and F2 (**B**) on human keratinocytes during UVB exposure (800 or 1200 J/m^2^). The viability of HaCaT cells was determined using Hoechst 33342 nuclei staining after incubation of cells for 24 h postirradiation. No cytotoxic effects were observed in treated nonirradiated cells. The data are expressed as the mean ± SD. *** (*p* < 0.001) or **** (*p* < 0.0001) indicate significant differences at the same UVB dose with nontreated cells, while ## (*p* < 0.01) or #### (*p* < 0.0001) indicate significant differences between cells treated with the other formulation at the same concentration with the same UVB irradiation dose.

At 800 J/m^2^, the presence of both formulations increased cell survival at all concentrations assayed (Figure 3). F1 and F2 protected HaCaT cells in a similar dose-dependent manner, reaching significant protection of 88.4% and 88.2%, respectively, at the maximum concentration used (200 µg/mL) when compared with irradiated cells in the absence of a formulation. However, differences between both formulation treatments were observed in keratinocytes exposed to a higher UVB dose (i.e., 1200 J/m^2^). At the minimum concentration (50 µg/mL), only F1 exerted a statistically significant protection, with a 27.3% cell survival increase. Although both formulations exhibited a dose-dependent behavior, F1 showed higher levels of photoprotection, by 68.5% at the highest concentration assayed, while 51.7% protection was achieved at the same concentration of F2.

### 3.2. Antioxidant Activity of the Formulations and Attenuation of ROS Generation in UVB-Irradiated HaCaT Cells

To examine the antioxidant capacity of both formulations, three different *in vitro* assays were performed (Table 2). Formulation F1 showed a significantly higher capacity to scavenge ABTS^•+^, showing a value of 417.6 ± 55.9 mmol TE/100 g dw in the TEAC assay, while 332.2 ± 56.5 mmol TE/100 g dw was determined for F2. Furthermore, ORAC measurements revealed that F1 also scavenged peroxyl radicals with more effectiveness than F2 (2638.8 ± 131.8 vs. 2114.0 ± 252.7 µmol TE/g dw, respectively). In contrast, F2 exhibited a higher ferric reducing ability when the FRAP assay was performed, obtaining values of 719.3 ± 71.2 mmol Fe^2+^/100 g dw for F1 and 857.7 ± 78.2 mmol Fe^2+^/100 g dw for F2. A comparison of both formulations reveals that this higher antioxidant capacity for F1 could be correlated to the statistically significant higher phenolic content determined for this formulation, i.e., 40.76 ± 2.59 GAE/100 g dw for F1 vs. 36.35 ± 2.72 GAE/100 g dw for F2.

**Figure 4:**
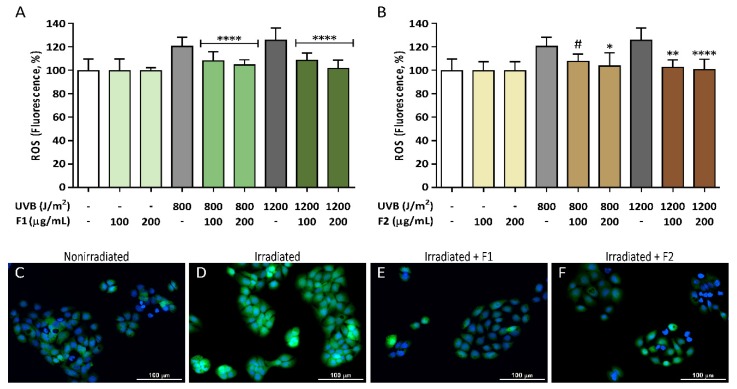
Inhibition of UVB-induced reactive oxygen species (ROS) generation in HaCaT keratinocytes exposed to UVB radiation (800 or 1200 J/m^2^) by formulations F1 (**A**) and F2 (**B**). Intracellular ROS generation was measured using a H_2_DCF-DA probe, and fluorescence was normalized to the nuclei number. Representative pictures of control nonirradiated HaCaT cells (**C**), control irradiated cells at 1200 J/m^2^ (**D**), irradiated cells at 1200 J/m^2^ in the presence of F1 (200 µg/mL) (**E**), and irradiated cells at 1200 J/m^2^ in the presence of F2 (200 µg/mL) (**F**) are shown. The scale bar represents 100 µm. The data are expressed as the mean ± SD. * (*p* < 0.05), ** (*p* < 0.01), or **** (*p* < 0.0001) indicate significant differences at the same UVB dose in irradiated and nontreated cells, while # (*p* < 0.05) indicates significant differences between cells treated with the other formulation at the same concentration with the same UVB irradiation dose.

The contribution of the antioxidant capacity of both formulations on their cellular protective properties against damaging radical species was evaluated in a cellular model after UVB irradiation. Intracellular ROS generation was monitored using the H_2_DCF-DA probe, which is oxidized to its fluorescent product by ROS, and fluorescence values were normalized to the cell nuclei number. Figure 4 shows increased intracellular ROS in UVB-exposed HaCaT cells compared to those cells that were not exposed to irradiation, which showed a basal ROS level. Intracellular UVB-induced ROS generation was inhibited in the presence of the formulations. At 800 J/m^2^ irradiation, the generated ROS significantly decreased by 75.8% after treatment with F1 and 80.4% for F2 at the 200 µg/mL concentration compared with their respective nonirradiated controls. Moreover, a higher effect was observed at 1200 J/m^2^ irradiation, with a statistically significant 92.4% ROS decrease for F1 and 96.3% decrease for F2 at the maximum concentration used. These percentages were calculated using the formula described in Section 2.5 where PC (positive control) was irradiated but nontreated sample fluorescence value and NC (negative control) was nonirradiated nontreated sample fluorescence value.

### 3.3. Influence of the Formulations on UVB-Induced Mitochondrial Depolarization

**Figure 5:**
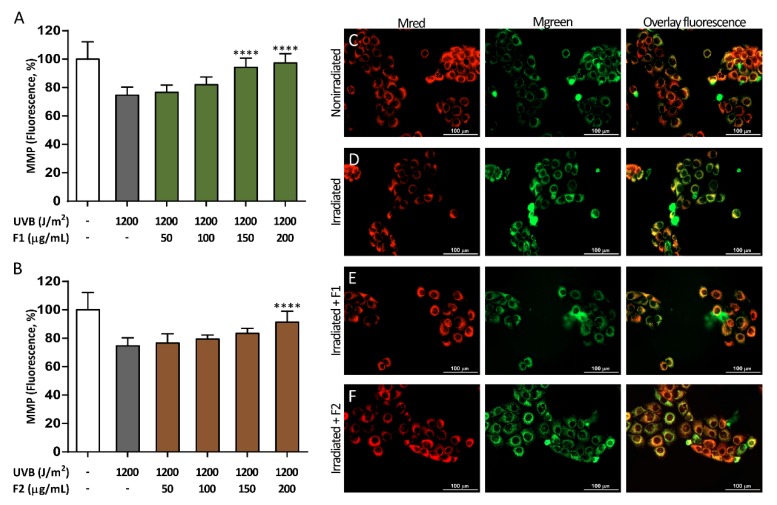
Mitochondrial membrane potential (MMP) restoration exerted by formulations F1 (**A**) and F2 (**B**) in HaCaT cells after UVB irradiation. MitoTracker Red CMXRos (Mred) and MitoTracker Green FM (Mgreen) were used to evaluate the MMP and to normalize to the total mitochondrial mass, respectively. Representative pictures of nonirradiated control cells (**C**), irradiated control cells at 1200 J/m^2^ (**D**), irradiated cells at 1200 J/m^2^ in the presence of F1 (200 µg/mL) (**E**), and irradiated cells at 1200 J/m^2^ in the presence of F2 (200 µg/mL) (**F**) are shown. The scale bar represents 100 µm. The data are expressed as the mean ± SD. **** (*p* < 0.0001) indicates significant differences between the irradiated and nontreated cells.

Mitochondrial membrane potential (MMP) is a marker of mitochondrial function, and two fluorescent probes (Mred and Mgreen) were used to analyze the MMP. Figure 5 shows the UVB-induced mitochondrial depolarization observed in keratinocytes exposed to UVB, which was revealed by decreased Mred staining without the loss of Mgreen staining. MMP was partially restored by both formulations with a dose-dependent trend. Nevertheless, statistically significant differences for F2 were observed only at the maximum concentration used, reaching a 65.8% mitochondrial function increase. In contrast, F1 exhibited a higher protection level, with 89.7% MMP restoration at the maximum concentration.

**Figure 6:**
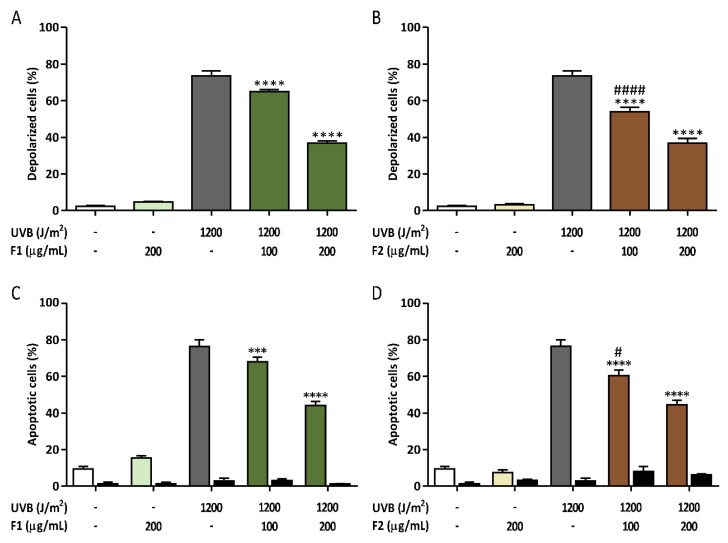
Reduction of UVB-induced mitochondrial depolarized HaCaT cells (**A**,**B**) and UVB-induced late apoptosis (**C**,**D**) by formulations F1 and F2. Black bars show early apoptosis. The data are expressed as the mean ± SD. *** (*p* < 0.001) and **** (*p* < 0.0001) indicate significant differences between irradiated and nontreated controls, while # (*p* < 0.05) and #### (*p* < 0.0001) indicate significant differences between irradiated cells treated with the other formulation at the same concentration. Representative population plots are included in the Supplementary Information, Figure S1.

In addition, mitochondrial viability was further studied using the Muse Cell Analyzer (Figure 6A,B) to confirm the results obtained with the MitoTracker probes. The percent of depolarized cells was close to 75% in the irradiated control cells, and this number was significantly reduced by 51.7% and 51.6% in the presence of 200 µg/mL F1 and F2, respectively, so both combinations exhibited a similar behavior.

### 3.4. Prevention of Late Apoptosis Detected in UVB-Irradiated HaCaT Cells by the Formulations

Apoptosis has an important function in the prevention of epidermal carcinogenesis by eliminating photodamaged cells. The percent of apoptotic cells was determined by staining with Annexin V kit described in materials section, which binds to exposed phosphatidylserine, using a Muse Cell Analyzer. UVB-induced cell damage initiates programmed cell death, and therefore, a high level of late apoptosis was shown in the nontreated irradiated cells 24 h post irradiation (Figure 6C,D). The presence of the two formulations significantly decreased the percent of apoptosis detected in a dose-dependent manner. Both formulations exhibited a similar protection level, reaching a statistically significant apoptotic cell reduction of 48.4% and 47.6% at the maximum concentrations used for F1 and F2, respectively. No influence on apoptosis was obtained for the maximum concentration (200 µg/mL) of either formulation in the absence of irradiation.

### 3.5. Influence of the Formulations on UVB-Induced DNA Damage

**Figure 7:**
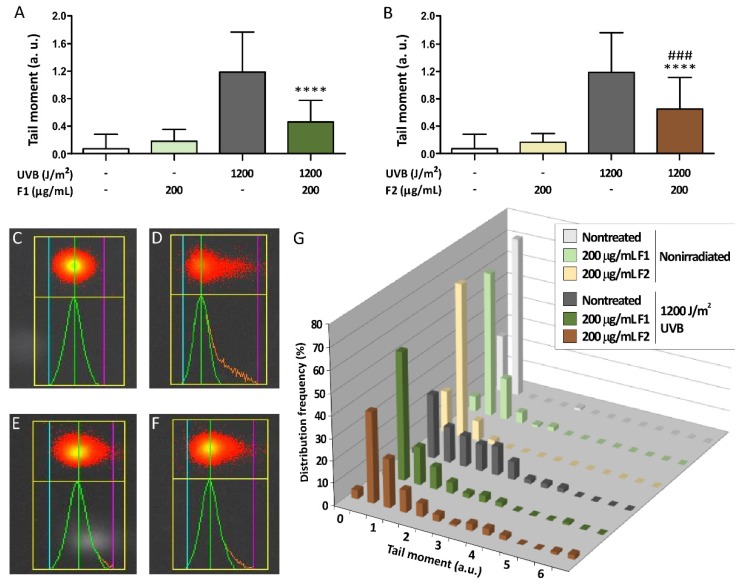
UVB-induced DNA double-strand break formation was inhibited by formulations F1 (**A**) and F2 (**B**) in irradiated human keratinocytes. The tail moment value (the product of the tail length and the tail DNA content) was automatically quantified using Comet Assay IV software. Representative comet pictures of the control of nonirradiated HaCaT cells (**C**), irradiated control cells at 1200 J/m^2^ (**D**), irradiated cells at 1200 J/m^2^ in the presence of F1 (200 µg/mL) (**E**), and irradiated cells at 1200 J/m^2^ in the presence of F2 (200 µg/mL) (**F**) are shown. The blue, green, and magenta lines indicate the start of the head, the center of the head and the end of the tail, respectively. The distribution frequency of tail moment values (**G**) was similar in nonirradiated cell populations and was altered due to UVB-induced DNA damage. The data are expressed as the mean ± SD. **** (*p* < 0.0001) indicates significant differences from the irradiated and nontreated controls, while ### (*p* < 0.001) indicates significant differences between irradiated cells treated with the other formulation.

To test whether the apoptosis reduction exerted by the formulations was due not only to the recovery of mitochondrial viability but also to a genoprotective effect of the formulations, the neutral comet assay was performed as a sensitive technique to detect DNA damage by the mobilization of free DNA-chromatin fragments associated with double-strand breaks (DSBs). Figure 7 shows representative pictures of the effects of the F1 and F2 formulations on the migrated DNA measured in HaCaT cells irradiated at 1200 J/m^2^. The quantitation of the tail moment values after treatment with both formulations in nonirradiated cells revealed that the tail moment value did not significantly change compared to the control cells, which suggests the absence of genotoxicity (Figure 7A,B). When cells were UVB-irradiated, the tail moment value increased considerably, and the frequency distribution of the population was altered (Figure 7G). The presence of 200 µg/mL F1 significantly reduced UVB-induced DNA damage by 64.9% compared with the untreated irradiated cells, while the F2 reduction was 47.9% at the same concentration.

**Figure 8:**
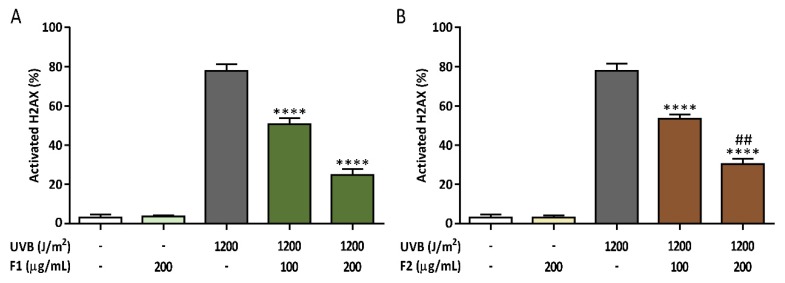
Formulations F1 (**A**) and F2 (**B**) decreased the DNA damage response in UVB-irradiated HaCaT cells by reducing H2AX histone activation. The data are expressed as the mean ± SD. **** (*p* < 0.0001) indicates significant differences compared with the irradiated nontreated control, while ## (*p* < 0.01) indicates significant differences between irradiated cells treated with the other formulation at the same concentration. Representative population plots are shown in the Supplementary Information, Figure S1.

To corroborate the lower level of UVB-induced DNA damage detected by the presence of the formulations, H2AX histone activation was evaluated by its phosphorylation as an early response to DNA damage. The phosphorylated form of H2AX (γ-H2AX) was measured in the absence or presence of the formulations in UVB-irradiated keratinocytes (Figure 8). As expected, a high percent of γ-H2AX was found in the irradiated control cells (close to 80%). Both formulations significantly decreased the γ-H2AX detected in a dose-response manner. F1 exhibited a higher genoprotective effect with a 70.8% γ-H2AX reduction at 200 µg/mL, while a 63.5% reduction was achieved when the same concentration of F2 was used.

## 4. Discussion

UV radiation, which is absorbed by the epidermis, is the major cause of a wide variety of cutaneous disorders, including photoaging and photocarcinogenesis. Numerous phytochemicals have shown the ability to protect the skin from the adverse effects of UVB radiation, including the risk of skin cancers. Concomitantly, significant interest in the generation of skin dietary supplements or topical application formulations based on botanicals with photoprotective properties and protection of their intellectual property is emerging [33].

In this study, two formulations containing citrus, olive, and rosemary extracts were investigated in order to evaluate their antioxidant capacity in correlation with their cellular protective properties against UVB-induced ROS generation. The strong antioxidant capacity was demonstrated for both formulations and could be correlated to their high phenolic content. Our results were in agreement with those previously reported after the use of similar botanical extracts [24,25], obtaining a similar level of cellular protection against UV radiation. When the antioxidant capacity of both formulations was compared, F1 showed a stronger ability to scavenge free radicals such as ABTS^•+^ and peroxyl radicals derived from the TEAC and ORAC assays, respectively, probably because of its higher content of citrus flavanone aglycones and the presence of diterpenes compared to F2. However, the FRAP assay revealed higher antioxidant properties for F2, which is consistent with the reported metal ion chelating capability of dihydroxylated flavones [34], which are present in F2 but not in F1. Both formulations exhibited a strong antioxidant ability, which confirms the potential of these compounds to inhibit the generation of intracellular damaging radical species induced by UVB radiation. Several plant phytochemicals have been shown to be efficient in preventing UV-induced oxidative stress through a ROS scavenging mechanism in vitro. Naringenin, a major flavonone aglycone constituent of grapefruit and other citrus extracts, has shown significant antioxidant and anti-inflammatory effects, and these molecular effects accounted for the improvement in antioxidant capacity in the skin [35]. Hydroxytyrosol, iridoids, and rosmarinic acid are abundant polyphenols in olive and rosemary extracts and have previously shown these protective effects [36,37].

Furthermore, the photoprotective capacity of the two formulations (F1 and F2) was evidenced in a skin cell model. Nontoxic concentrations of formulations F1 and F2 exhibited the ability to increase cell survival of UVB-exposed keratinocytes in our experiments with a dose-dependent trend. The F1 formulation, that contained the rosemary extract, exhibited a stronger protective capacity through the increase in cell survival when cells were exposed to a higher UVB dose (i.e., 1200 J/m^2^). Therefore, we postulate that rosemary compounds may contribute to the stronger photoprotective effects of F1. Carnosic acid and carnosol are the most important active components of rosemary extracts [38]. These diterpenes may improve the protective capacity of F1 due to the strong capacity to scavenge the lipid peroxyl radicals described for these compounds, which cause DNA damage and initiate inflammatory processes [39]. The highest ORAC value, which was obtained for F1, strengthened our hypothesis. Our results also indicate that the F1 formulation had a stronger genoprotective capacity than the F2 formulation since it more efficiently inhibited DNA damage, as seen by the comet assay and H2AX activation, as well as stronger protection of mitochondrial viability. The capacity of carnosic acid to prevent photoaging through the reduction of UVB-enhanced GADD45 expression, a marker for oxidative DNA damage, and the decrease of UVB-induced expression of matrix metalloproteinases in human skin cells has been reported [40]. Furthermore, we postulate that the stronger genoprotective capacity of F1 vs. F2 may also be related not only to the stronger radical scavenging capability of flavanone aglycones compared to their glycosides but also to the capacity of flavanone aglycones to promote DNA repair. In this regard, the flavanone aglycone naringenin has shown a DNA repair-stimulating capacity through the upregulation of several enzymes involved in DNA base excision repair [41].

To investigate the possible causes that may account for the observed photoprotective effects, the absorption spectra of the formulations were analyzed and compared to detect a putative barrier effect. The spectra of both formulations showed an absorption maximum at 280 nm within the UVB region, which is typical of benzoyl systems [42]. In addition, F1 exhibited a shift of the second maxima downwards to 330–335 nm that is typical of a B-ring monohydroxylated flavone moiety within the UVA region, which is probably due to its higher content in these compounds. The stronger photoprotective capacity shown in the cell survival assay using UVB radiation (290–320 nm) for the F1 formulation might be due, in part, to the presence of strong spectral absorption of the compounds contained in this formulation within the UVB range leading to the blockade of radiation. Nevertheless, since some bioactive compounds may rapidly reach intracellular targets [43], we propose that the compounds in the formulations may be concomitantly able to scavenge the above described ROS, which subsequently cause oxidative DNA, lipid, and protein damage.

Intracellular ROS also act as inflammatory mediators through the induction of the expression of proinflammatory enzymes and cytokines such as interleukin (IL)-1β, IL-6, IL-9, and cyclooxygenase-2 (COX2) [19]. In fact, the overproduction of ROS mediates the activation of NF-κB transcription factors, which are of central importance in inflammation [44]. In this context, we postulate that both formulations might be capable of reducing the ROS-induced inflammatory response by modulating the nuclear factor kappa-light-chain-enhancer of activated B cells (NF-κB) signaling pathway, as other polyphenolic extracts have demonstrated the same molecular effects [45]. This hypothesis is in agreement with previous findings that showed the ability of naringenin to reduce the UVB-induced production of several inflammatory cytokines and the expression of gp91phox mRNA, which is the NADPH oxidase 2 (NOX2) subunit responsible for generating O_2_^•−^ [46]. This is also consistent with the anti-inflammatory capacity of olive iridoids or carnosol through the reduction of NF-κB subunit translocation [47,48].

Mitochondria are also an important source of ROS within most mammalian cells through the mitochondrial electron transport system. Increased intracellular ROS induces mitochondrial depolarization and dysfunction that can be used as a biomarker for oxidative stress [49]. In our experiments, both formulations showed a mitochondrial protective effect by restoring the MMP and diminishing the percent of depolarized cells. Several in vitro and in vivo studies have demonstrated that flavans such as epigallocatechin-3-gallate (EGCG) from green tea or the flavanone naringenin protect mitochondria at the molecular level [50]. It has been postulated that these compounds reduce oxidative stress and improve mitochondrial function via activation of the nuclear factor-erythroid 2-related factor 2 and antioxidant responsive element (Nrf2-ARE) signaling pathway [51,52]. The transcription factor Nrf2 is the major factor responsible for the regulation of ARE-driven expression of genes encoding important detoxifying and antioxidant enzymes. It has been observed that UVB radiation significantly decreases Nrf2 mRNA expression [53]. Therefore, this transcription factor might be involved in the photoprotective action of the formulations. Naringenin has also been shown to inhibit the UVB-induced reduction of Nrf2 mRNA expression in mouse skin [35]. Hydroxytyrosol from olive extract and carnosic acid have also been shown to increase Nrf2 expression in other cell lines [54,55]. Furthermore, formulations F1 and F2 showed a significant reduction in the percent of late apoptosis detected after 24 h in UVB-irradiated keratinocytes. Mitochondria are involved in one of the two major apoptosis signaling pathways (active and passive). The release of mitochondrial cytochrome *C* activates the caspase signaling cascade and promotes cell death [56]. Therefore, we hypothesize that the formulations may suppress mitochondria-mediated apoptosis by preventing ROS generation and MMP alteration. In this regard, the capacity of the flavone luteolin to inhibit MMP alteration and increase the resistance of normal, but not malignant, keratinocytes against UVB-induced apoptosis has been reported [57]. Furthermore, naringenin treatment protected UVB-exposed HaCaT cells from apoptosis by affecting the caspase pathway [58]. Since both formulations contain flavanones and flavones, both formulations seem to be capable of preventing UVB-induced mitochondrial depolarization and apoptotic cell death in a similar manner.

In addition, direct and indirect effects of UVB irradiation on DNA lead to mutations or genome aberrations and even cell death. Cells have evolved repair pathways to detect DNA lesions and promote their repair through the DNA damage response (DDR). Key mediators of the DDR are the ATM (ataxia telangiectasia mutated) and ATR (ataxia telangiectasia and Rad-3 related) kinases, which phosphorylate serine 139 of the histone H2A variant H2AX when activated by damaged DNA [59]. Activated H2AX (γ-H2AX) acts as a sensor that coordinates DNA repair with cell cycle checkpoint control through the recruitment of DDR factors plus other chromatin-modifying components. If DNA damage is not repaired and DDR persists, apoptosis is usually mediated by p53 and checkpoint kinase 1 (CHK1). It has been shown that UVB irradiation induces H2AX activation, and DDR proteins are recruited to CPD-damaged DNA sites [60]. Additionally, it has been elucidated that ATM phosphorylates H2AX in response to DSBs [61]. According to our results, the presence of both formulations promotes a significant decrease in DNA damage in UVB-exposed keratinocytes by preventing DSB formation detected in the neutral comet assay, which has been corroborated with the minor percentage of phosphorylated H2AX detected. Moreover, F1 showed a lower level of DNA damage in the two mentioned assays. In this sense, the capacity of flavanone aglycones such as naringenin to enhance the removal of CPD lesions from the genome of HaCaT cells has been reported [58]. Of note, oxidative stress-driven lipid peroxidation is also responsible for continuous DNA damage [62].

The diterpenes carnosic acid and carnosol have exhibited the capacity to prevent the generation of radical species that are caused by both ionizing and nonionizing radiation [63,64]. It has been shown that particular ROS species, such as singlet oxygen (^1^O_2_), hydroxyl radicals (^•^OH) and lipoperoxy radicals (R–OO·), are those most likely to cause photoinduced DNA and chromosomal oxidative damage upon UV radiation. Some of these lesions, such as single oxidized bases, are in most cases efficiently removed through base excision repair. In contrast, dimeric lesions triggered by both UVA and UVB radiation, such as CPDs and pyrimidine (6-4) pyrimidone photoproducts (6-4PPs), may lead to severe DNA damage if they are not efficiently removed by nucleotide excision repair [65].

In conclusion, the two formulations, F1 and F2, exerted photoprotective effects against UVB radiation in the cellular skin model. Both formulations showed the ability to increase the cell survival of UVB-exposed keratinocytes, while ROS generation, MMP changes, and DNA damage were diminished. Furthermore, we found stronger genoprotective effects from F1, and we postulate that not only rosemary diterpenes but also some flavanone aglycones may contribute to the stronger protective capacity of F1 compared to that of F2. However, the stronger potency of F2 in the FRAP assay may be useful to inhibit the generation of metal-related free radicals and the subsequent oxidative damage. Whole extracts are definitely more convenient and less expensive for nutraceutical or cosmetic applications than isolated compounds. Nevertheless, when these formulations are used as nutraceuticals instead of as topical agents, metabolic transformations of the polyphenols should be considered through human trials in order to determine the final metabolites targeting skin cells.

## Figures and Tables

**Table 1:** Analytical composition in bioactive compounds (dry weight) for the formulations F1 and F2 as declared by the manufacturer. Both formulations were composed of citrus and olive extracts while F1 also contained rosemary extract.

		Dry Weight Content (%)	
		F1	F2
	Iridoids	15	15
	Diterpenes	5	0
Flavanones	Aglycones	33	0
	Glycosides	12	50
Flavones	Monohydroxylated aglycones	34	17
	Dihydroxylated aglycones	0	17
	Glycosides	1	1

**Table 2:** Phenolic content and values for different antioxidant measurements performed with the formulations F1 and F2.

Assay	F1	F2	Student’s t-Test
Folin-Ciocalteu (g GAE ^a^/100 g dw ^c^)	40.8 ± 2.6	36.4 ± 2.7	####
TEAC (mmol TE ^b^/100 g dw ^c^)	417.6 ± 55.9	332.2 ± 56.5	##
ORAC (μmol TE ^b^/g dw ^c^)	2638.8 ± 131.8	2114.0 ± 252.7	####
FRAP (mmol Fe^2+^/100 g dw ^c^)	719.3 ± 71.2	857.7 ± 78.2	#

Data are expressed as mean ± SD. ^a^ Gallic acid equivalents, ^b^ Trolox equivalents, ^c^ dry weight. # (*p* < 0.05), ## (*p* < 0.01) or #### (*p* < 0.0001) indicate significant differences between both formulations.

## Supplementary Materials

The following are available online at https://www.mdpi.com/2076-3921/9/3/255/s1: Figure S1: Representative population plots of depolarized cells (A–D), apoptotic cells (E–H), and activated H2AX (I–L) obtained using the Muse Cell Analyzer. There are included plots from control of nonirradiated HaCaT cells (A,E,I), control of irradiated cells at 1200 J/m^2^ (B,F,J), irradiated cells at 1200 J/m^2^ in the presence of F1 (200 µg/mL) (C, G, K), and irradiated cells at 1200 J/m^2^ in the presence of F2 (200 µg/mL) (D,H,L).
