# Supplementary material for: Rosemary Diterpenes and Flavanone Aglycones Provide Improved Genoprotection against UV-Induced DNA Damage in a Human Skin Cell Model
Source: Antioxidants (Basel). 2020 Mar 20;9(3):255. doi: 10.3390/antiox9030255 (PMC7139908; doi:10.3390/antiox9030255)
Supplement: Supplementary file 1 [file antioxidants-09-00255-s001.pdf]

Rosemary diterpenes and flavanone aglycones provide improved genoprotection against UV-induced DNA damage in a human skin cell model

Noelia Sánchez-Marzo[1], Almudena Pérez-Sánchez[1], Enrique Barrajón-Catalán[1, #], Julián Castillo[2], María Herranz-López[1†], and Vicente Micol[1, 3, †]

[1.] Instituto de Investigación, Desarrollo e Innovación en Biotecnología Sanitaria de Elche (IDiBE), Instituto de Biología Molecular y Celular (IBMC), Miguel Hernández University (UMH), 03202 Elche, Spain; n.sanchez@umh.es (N.S.-M.); almudena.perez@umh.es (A.P.-S); e.barrajon@umh.es (E.B.-C.); mherranz@umh.es (M.H.-L) vmicol@umh.es (V.M.)

[2] Nutrafur S.A., Camino Viejo de Pliego, km.2, 30820 Alcantarilla, Murcia, Spain; Department of Food Technology and Nutrition, Universidad Católica San Antonio, Murcia, Spain. j.castillo@nutrafur.com (J.C.)

[3] CIBER, Fisiopatología de la Obesidad y la Nutrición, CIBERobn, Instituto de Salud Carlos III (CB12/03/30038), Spain

†These authors shared author co-seniorship.

[#] *Corresponding author:* e.barrajon@umh.es

Supplementary information

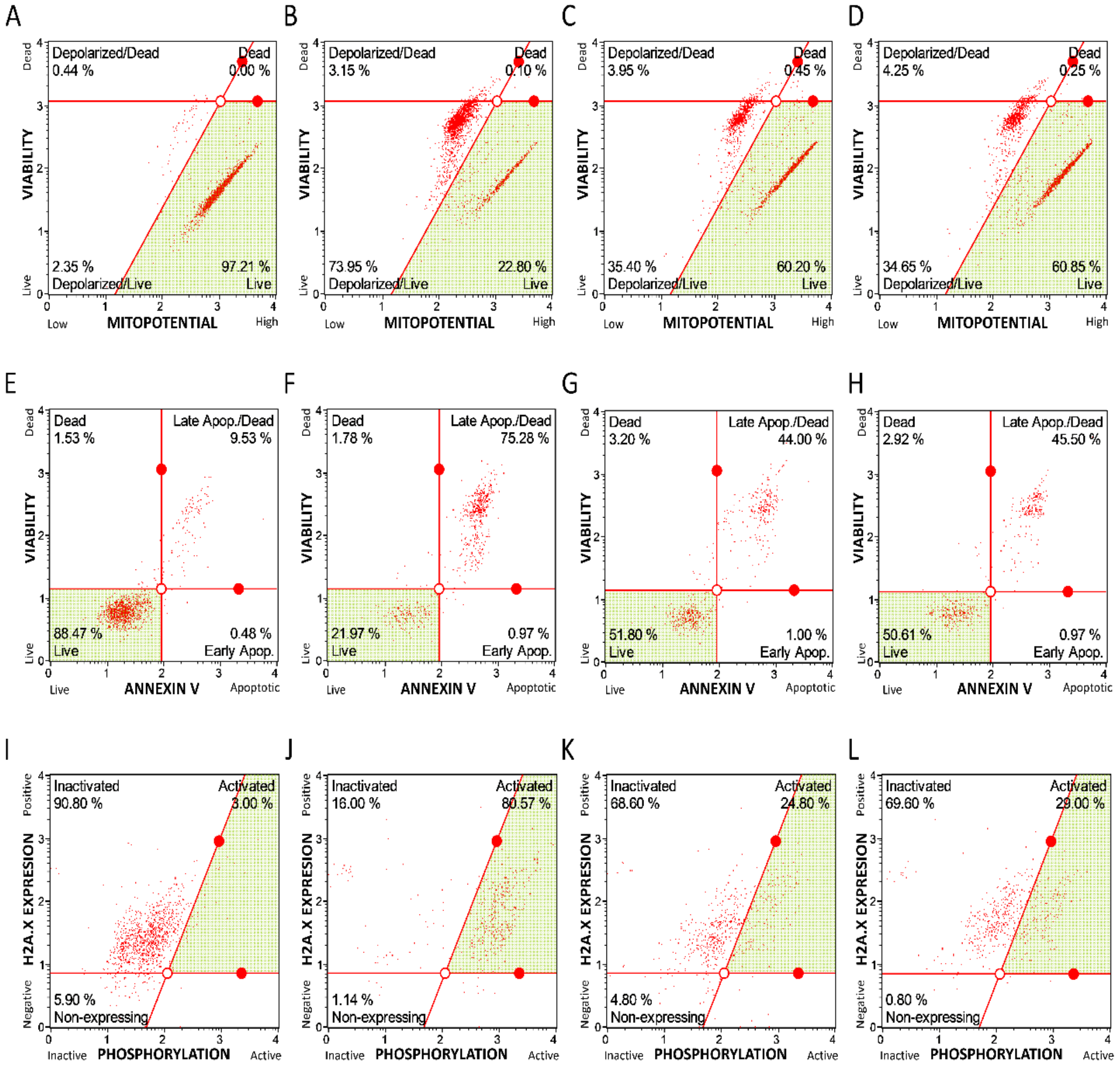

Figure S1. Representative population plots of depolarized cells (A-D), apoptotic cells (E-H) and activated H2AX (I-L) obtained using the Muse Cell Analyzer. There are included plots from control of nonirradiated HaCaT cells (A, E, I), control of irradiated cells at 1200 J/m^2 (B, F, J), irradiated cells at 1200 J/m^2 in the presence of F1 (200 μg/mL) (C, G, K) and irradiated cells at 1200 J/m^2 in the presence of F2 (200 μg/mL) (D, H, L).
